# Tomato HAIRY MERISTEM4, expressed in the phloem, is required for proper shoot and fruit development

**DOI:** 10.1093/hr/uhae325

**Published:** 2024-11-21

**Authors:** Jackson Khedia, Abhay Pratap Vishwakarma, Ortal Galsurker, Shira Corem, Suresh Kumar Gupta, Tzahi Arazi

**Affiliations:** Institute of Plant Sciences, Agricultural Research Organization, Volcani Center, 68 HaMaccabim Road, P.O.B 15159 Rishon LeZion 7505101, Israel; Department of Botany, Deshbandhu College, University of Delhi, Kalkaji Main Rd, Block H, Kalkaji, New Delhi, Delhi 110019, India; Institute of Plant Sciences, Agricultural Research Organization, Volcani Center, 68 HaMaccabim Road, P.O.B 15159 Rishon LeZion 7505101, Israel; BetterSeeds Ltd., Birkat Am 54, Givat Hen 43905, POB 46, Rishon LeZion, Israel; Department of Biochemistry and Molecular Biology, Michigan State University, Biochemistry building 603 Wilson Road, Room 310A, East Lansing, Michigan, 48824, USA; Institute of Plant Sciences, Agricultural Research Organization, Volcani Center, 68 HaMaccabim Road, P.O.B 15159 Rishon LeZion 7505101, Israel

## Abstract

The HAIRY MERISTEM (HAM) gene family encodes Type I and II GRAS domain transcriptional regulators in plants. Type II HAMs, predominantly expressed in meristems and regulated by microRNA171, are essential for maintaining undifferentiated meristems, a role conserved across various species. Conversely, the functions of Type I HAMs have been less characterized. In this study, we investigated the role of SlHAM4, a Type I HAM in tomato. CRISPR-induced *SlHAM4* loss-of-function mutations (*slham4*^***CR***^) resulted in shoot and fruit abnormalities, which were fully reversed by reintroducing *SlHAM4*, driven by its native promoter, into the mutant background. Mutant abnormalities included simpler leaves and increased anthocyanin pigmentation in the leaf and sepal primordia, reminiscent of phenotypes observed in certain Arabidopsis mutants with compromised phloem. In addition, *slham4*^***CR***^ plants produced significantly smaller fruits with a subset developing catface-like scars, attributed to tears that occurred in the pericarp of setting fruits. Using a GUS reporter gene driven by the native *SlHAM4* promoter, we found that *SlHAM4* is predominantly expressed in phloem tissues. Consistent with this, transcriptome analysis of mutant anthesis ovaries revealed specific downregulation of genes implicated in phloem development and function, particularly those expressed in companion cells. However, histological analysis showed no obvious abnormalities in phloem vasculature. Taken together, our data suggest that SlHAM4 plays a role in shoot and fruit development likely by regulating genes essential for phloem function.

## Introduction

The plant HAIRY MERISTEM (HAM) gene family, initially identified in Petunia [1] encodes GRAS (GAI, RGA, and SCR) domain transcriptional regulators. In Arabidopsis, four HAM homologs (HAM1–HAM4) have been identified and are categorized into two groups, Type I and Type II, according to phylogenetic analysis [2]. The Petunia *ham* mutant exhibits hairy shoot apical meristem (SAM) resulting from its premature differentiation, as well as termination of lateral organ development [1].

In Arabidopsis, the Type II HAM group includes HAM1, HAM2, and HAM3, also known as LOST MERISTEM1 (LOM1), LOM2, and LOM3. These genes are primarily expressed in shoot and root meristems and in provascular tissues [2, 3]. In addition, they are subject to negative regulation by microRNA171 (miR171), which mediates transcript cleavage, thereby modulating gene expression [4]. Individual loss-of-function mutations in these genes generally do not cause major developmental anomalies. However, combined mutations or miR171 overexpression result in significant phenotypic changes. These include early SAM termination and reduced axillary shoot branching, associated with disorganized shoot apical and axillary meristems [2, 3]. Further analysis of mutants shows that HAM1 and HAM2 primarily contribute to the maintenance of undifferentiated SAMs and the initiation of new axillary stem cell niches. In contrast, HAM3 plays a minor role in maintaining SAM, and is involved in axillary meristem development [5]. Notably, HAM1 and HAM2 directly interact with the WUSCHEL (WUS) protein, functioning as transcriptional cofactors. In addition, they prevent *CLAVATA3* (*CLV*3) expression within the inner cells of the SAM. These activities are pivotal in controlling shoot stem cell production and maintaining the indeterminacy of the SAM. In several flowering plants, the *HAM* gene family maintains indeterminate SAMs and promotes new axillary meristem formation, suggesting that this function is conserved across various plant species [6].

Type I *HAM* genes show variation in their miR171 target sequences, unlike Type II *HAM* genes. Some Type I *HAM* genes retain the conserved miR171 binding sequence, while others, such as *HAM4* in Arabidopsis, have lost it [2, 4], suggesting potential functional diversification. The Arabidopsis Type I gene *HAM4* (also referred to as *SCARECROW-LIKE15*) is expressed in the vasculature of leaves, roots, stems, and siliques, particularly in phloem tissues, including companion cells [3, 7]. HAM4 has been shown to physically interact with WUSCHEL-RELATED HOMEOBOX4 (WOX4), which is expressed in procambial cells that define the vascular stem cell niche. This interaction, coupled with their coinciding expression patterns, suggests HAM4 may act as a cofactor for WOX4 [3]. However, phenotypic analysis of *scl15/ham4* mutants revealed no significant abnormalities in plant growth, except for being slightly smaller and showing a minor delay in flowering [3, 7]. In addition, HAM4/SCL15 has been identified as a partner of HISTONE DEACETYLASE19 (HDA19) in Arabidopsis seedlings. Microarray analysis of mutant *scl15/ham4* seedlings revealed that loss of *HAM4/SCL15* led to significant changes in gene expression, with numerous seed maturation genes being upregulated in vegetative tissues. The upregulation of a fraction of them was associated with increased histone acetylation, an epigenetic mark linked with expression activation. These results suggest that SCL15 acts as an HDA19-interacting regulator, repressing a subset of seed maturation genes through histone deacetylation [7]. Nevertheless, whether HAM4 plays specific roles in phloem development and function remains largely elusive.

**Figure 1:**
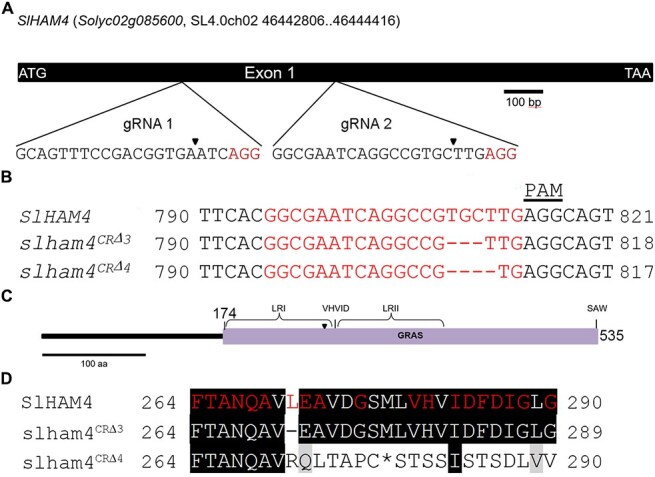
CRISPR/Cas9-mediated mutagenesis of the *SlHAM4* gene. (**A**) Schematic of the *SlHAM4* gene structure with gRNA target sites marked. Expanded views show gRNA target sequences with PAM motifs (red); arrowheads indicate expected Cas9 cleavage sites. (**B**) Alignment of CRISPR mutant alleles (*slham4^CR^*) with the wild-type *SlHAM4* sequence. gRNA target sequences are highlighted in red, and the PAM motif is noted. Numbering is from the start codon. (**C**) Representation of SlHAM4 protein architecture. The GRAS domain, key sequence motifs and regions, are shown. The conserved Leu^271^ location is marked with an arrowhead. (**D**) Alignment of mutant and wild-type SlHAM4 protein sequences. Amino acids in the GRAS domain conserved across species are highlighted in red, according to the NCBI Conserved Domain Database (https://www.ncbi.nlm.nih.gov/cdd/). Numbering starts from the start codon, with an asterisk (*) indicating a premature stop codon.

Tomato has three HAM homologs: the Type II SlHAM and SlHAM2, and the Type I SlHAM4 (Solyc02g085600). *SlHAM* and *SlHAM2* are targeted by miR171 and are abundant in shoot and floral meristems and compound leaf primordia. Silencing *SlHAM* and *SlHAM2* causes overproliferation of stem cells in the meristem periphery and the organogenic compound leaf rachis. This suggests that they have conserved roles in meristem maintenance [8]. Unlike *SlHAM* and *SlHAM2*, *SlHAM4* is not targeted by miR171 and is practically absent from meristems and compound leaf primordia [8], suggesting that it is not playing a major role in meristem maintenance and may have other functions.

In this study, we investigated the role of SlHAM4 in tomato. Unlike Arabidopsis *scl15/ham4* mutants [3, 7], CRISPR/Cas9-induced loss-of-function mutations in *SlHAM4* (*slham4^CR^*) led to shoot and notable fruit abnormalities, including smaller fruits with catface-like scars. These defects were completely rescued by reintroducing *SlHAM4* under its native promoter, confirming that the abnormalities were due to the loss of SlHAM4 function. Similar to Arabidopsis *SCL15/HAM4* [3, 7], our results indicate that *SlHAM4* is predominantly expressed in phloem tissues. Supporting this, *slham4^CR^* ovaries at anthesis showed downregulation of phloem-associated genes, although phloem vasculature appeared normal. Overall, our results suggest that *SlHAM4* is required for shoot and fruit development, likely by regulating the expression of genes essential for phloem functions in corresponding tissues.

## Results

### The *slham4^CR^* mutations are associated with abnormal shoot and fruit development

To functionally characterize *SlHAM4*, we first employed CRISPR/Cas9 technology to knock out its gene (Fig. 1a). This strategy yielded two distinct *SlHAM4* mutant alleles with deletions of three and four base pairs, named *slham4^CRΔ3^* and *slham4^CRΔ4^*, respectively (Fig. 1b). The SlHAM4 protein is a member of the GRAS family of transcriptional regulators [9]. It is characterized by a variable N-terminus and a highly conserved GRAS domain, which includes leucine-rich regions (LRI and LRII) surrounding a VHIID motif (V^280^HVID^284^) and a C-terminal SAW motif (S^532^AW^534^) (Fig. 1c). The *slham4^CRΔ3^* mutation is predicted to cause the loss of the conserved Leu^271^ within the LRI region (Fig. 1c and d). The *slham4^CRΔ4^* mutation introduces a premature stop codon, resulting in a truncated SlHAM4 protein missing a substantial portion of its GRAS domain (Fig. 1d), suggesting that it represents a loss-of-function mutation.

**Figure 2:**
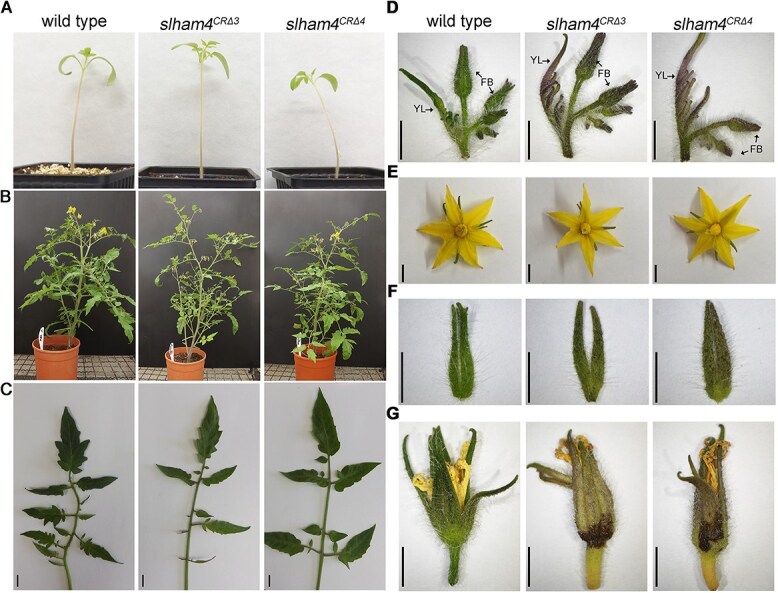
Comparison of M82 (wild-type) and *slham4^CR^* shoot and flower phenotypes. (**A**) Seedlings at 14 days post-germination. (**B**) Mature plants cultivated in standard greenhouse conditions. (**C**) A representative fully expanded leaf. (**D**) Representative young leaves and inflorescences at the sympodial shoot. YL, young leaf; FB, flower bud. Note the increased purple pigmentation of mutant young leaves and sepals. (**E**) Anthesis flowers. (**F**) Detached sepals from anthesis flowers. (**G**) Representative flowers not subjected to fertilization, displaying senescence; mutant sepals exhibit noticeable dark brown areas. Scale bars = 1 cm (**C**) and 5 mm (**D**–**G**).

To investigate the role of *SlHAM4* in tomato development, we compared the vegetative and reproductive phenotypes of the *slham4^CR^* mutants to the M82 parental line (hereafter referred to as wild type). Initially, both *slham4^CRΔ3^* and *slham4^CRΔ4^* mutants exhibited normal germination and seedling growth, similar to wild-type seedlings (Fig. 2a). However, as they matured, the mutants could be distinguished from wild-type plants based on the reduced density of their shoots, due to the compound leaves of the mutants bearing fewer leaflets (Fig. 2b and c). Furthermore, the *slham4^CR^* mutants displayed pronounced purple pigmentation in leaf primordia and flower bud sepals, indicating anthocyanin accumulation, a phenotype never observed in wild-type plants under identical growth conditions (Fig. 2d). The purple pigmentation was also present in the sepals of *slham4^CR^* flowers at anthesis, although no other differences compared to wild-type flowers were observed (Fig. 2e and f). Unfertilized wild-type tomato flowers senesce approximately 5 days after anthesis, marked by petal dehydration and weak chlorosis of sepals. While petal dehydration occurred in senescing *slham4^CR^* mutant flowers, their sepals exhibited strong chlorosis and browning of certain areas at their base, deviating from the typical sepal senescence pattern observed in wild-type plants (Fig. 2g).

**Figure 3:**
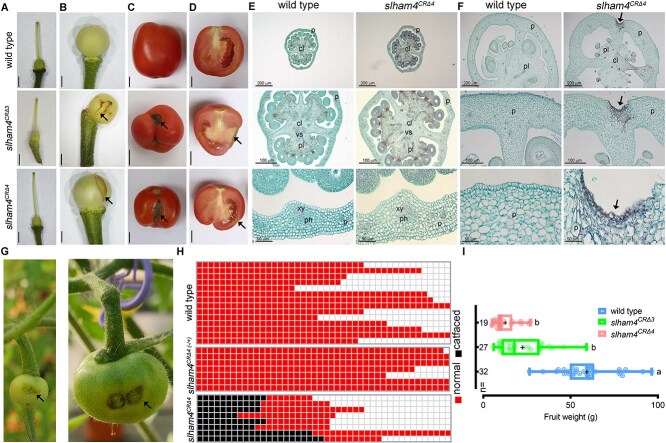
Characterization of M82 (wild-type) and *slham4^CR^* fruits. (**A**) Isolated pistils from anthesis flowers. (**B**) Young immature green fruits. Arrows mark the pericarp ruptures in mutants. (**C** and **D**) Red ripe fruits and their longitudinal sections, respectively. Arrows point to catface-like scars. Scale bars = 2 mm (**A** and **B**) and 1 cm (**C** and **D**). (**E** and **F**) Cross-sections of anthesis ovaries and 4-mm stage young immature green fruits, respectively, stained with safranin and fast-green, where the red staining delineates xylem vasculature. The middle and bottom panels show close-up views of respective upper panel. p, pericarp; cl, columella; pl, placenta; vs, vascular tissue; xy, xylem; ph, phloem. In **F**, arrows mark the indentation in the mutant fruit pericarp. (**G**) Young immature and mature green wild-type fruits developed from anthesis ovaries with manually crushed pericarp, resulting in a ~1 mm indentation. The resulting catface-like scars are indicated by arrows. (**H**) Diagram illustrating the prevalence of catface formation in fruits from wild-type, heterozygous (*slham4^CRΔ4−/+^*), and homozygous (*slham4^CRΔ4^*) plants, with each row representing an individual plant. Plants were cultivated in greenhouse nested plots. (**I**) Average fruit weight of indicated genotypes. The median and average are indicated by a line and +, respectively; n = number of fruits; Different letters indicate significance (*P* < 0.01) as determined by Tukey–Kramer multiple comparison test.

At anthesis, the *slham4^CR^* flower pistils appeared morphologically identical to wild-type controls (Fig. 3a). Despite the relatively high *SlHAM4* expression in the placenta and columella within the ovary (Fig. S1a–c), histological analysis revealed no obvious differences in the overall structure or vasculature arrangement between wild-type and mutant ovaries at anthesis (Fig. 3e). However, we noticed that following fruit set, a fraction of *slham4^CR^* young immature green fruits exhibited small tears in their pericarp (Fig. 3b). As the fruit grew, these tears expanded, eventually exposing the locules to air, resulting in scarring (Fig. 3c and d). Histological analysis of *slham4^CRΔ4^* young immature green fruits revealed indentations in the pericarp containing collapsed cells (Fig. 3f). We hypothesized that the inability of the damaged tissue to grow with the rest of the pericarp resulted in a tear that expanded as the fruit developed. To test this, we created a ~1mm indentation in the pericarp of wild-type anthesis ovaries by gently crushing it with the tip of a pencil. This led to the formation of a tear that expanded as the fruit grew (Fig. 3g), suggesting that localized pericarp damage can lead to significant fruit scars. Fruit scars in *slham4^CR^* mutants varied in size and shape (Fig. 3c and Fig. S2a–c). This fruit pericarp scarring phenotype is highly reminiscent of the tomato fruit catfacing syndrome [10, 11]. Notably, catfacing was observed in many, but not all, *slham4^CRΔ4^* fruits, suggesting incomplete penetrance. Under our greenhouse conditions, 30%–70% of *slham4^CRΔ4^* fruits displayed catfacing, while both heterozygous *slham4^CRΔ4(−/+)^* and wild-type fruits were completely devoid of catfacing (Fig. 3h). Mutant fruits with and without catfacing ripen normally, and their internal morphology resembled that of wild-type tomatoes (Fig. 3c and d), though their final size was significantly smaller than wild-type fruits (Fig. 3i).

### *SlHAM4* transgenic expression reverts mutant phenotypes and delineates its promoter activity

**Figure 4:**
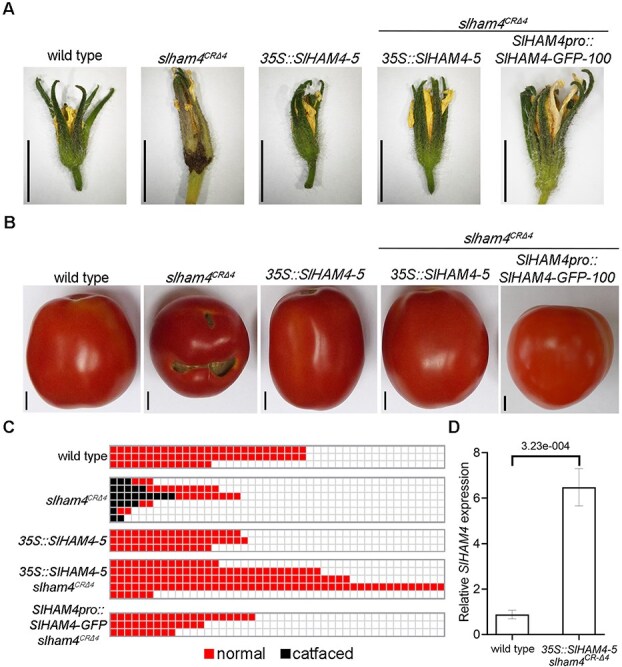
Functional complementation of *slham4^CRΔ4^* mutation. (**A** and **B**) Images of representative senescing flowers (**A**) and red ripe fruits (**B**) from M82 (wild type) and indicated genotypes. Scale bars = 1 cm. (**C**) Schematic representation of catface development in fruits of indicated genotypes. Each column represents a single plant. (**D**) Quantitation of *SlHAM4* transcript levels in M82 (wild-type) and *35S::SlHAM4–5 slham4^CRΔ4^* anthesis ovaries, normalized to *SlTIP41* as the reference gene. Error bars indicate ±SD over three biological replicates. The *P*-value as determined by Student’s *t*-test is shown.

To confirm the specific contribution of *SlHAM4* to the observed *slham4^CR^* phenotypes, we investigated whether reintroducing a functional copy could reverse the effects of the loss-of-function mutation. First, we generated transgenic M82 plants expressing *SlHAM4* driven by the constitutive cauliflower mosaic virus *35S* (*35S*) promoter (Fig. S3a). Among the regenerated lines, *35S::SlHAM4–5* showed the highest level of *SlHAM4* overexpression (Fig. S3b) with no visible or developmental abnormalities (Fig. S3c and Fig. 4a and b). Crossing this line with the homozygous *slham4^CRΔ4^* mutant and analyzing the *35S::SlHAM4–5 slham4^CRΔ4^* F2 progeny revealed no difference in flower senescence and fruit development compared to wild type (Fig. 4a–c). The elimination of fruit catfacing coincided with a 6-fold increase in *SlHAM4* expression in *35S::SlHAM4–5 slham4^CRΔ4^* anthesis ovaries compared to wild type (Fig. 4d).

The *SlHAM4* gene is situated on chromosome two, located 8.82 kb upstream of *Solyc02g085590* and 0.48 kb downstream of *Solyc02g085610* (Fig. S3d). To delineate the *SlHAM4* endogenous promoter, we generated transgenic *slham4^CRΔ4^* plants expressing a SlHAM4-GFP fusion protein driven by a 4 kb fragment of the putative promoter located upstream of the *SlHAM4* start codon (Fig. S3d and e). Among regenerated transgenic lines, *SlHAM4pro::SlHAM4-GFP slham4^CRΔ4^* lines 8, 100, and 102 regained normal flower senescence and were completely devoid of fruit catfacing, indicating sufficient activity of the 4 kb promoter fragment (Fig. 4a–c). Collectively, these experiments strongly support a direct link between SlHAM4 function and the observed phenotypes in *slham4^CR^* mutants. Moreover, the similar phenotypic abnormalities between *slham4^CRΔ3^* and *slham4^CRΔ4^* mutants (Fig. 2 and Fig. 3) reinforce the classification of *slham4^CRΔ3^* as a loss-of-function allele, highlighting the importance of the conserved Leu^271^ residue in the GRAS domain for the proper function of SlHAM4.

### *SlHAM4* is predominantly expressed in the phloem

To explore the tissue-specific expression pattern of *SlHAM4*, we initially queried public tomato databases for *SlHAM4* expression. These revealed that in seedlings, *SlHAM4* is predominantly expressed in the shoot compared to the root (Fig. S1a inset). In mature plants, *SlHAM4* is expressed in both vegetative and reproductive tissues, with the highest expression levels detected in anthesis flowers and orange fruit pericarp (Fig. S1a). In the anthesis flower ovary, *SlHAM4* expression is strongest in the placenta and columella, tissues known for their rich vasculature [12] (Fig. S1b and c). In developing and ripening fruits, *SlHAM4* is predominantly expressed in the pericarp vasculature and its levels increase progressively throughout fruit development and ripening, peaking in orange and red fruits (Fig. S1b–d).

**Figure 5:**
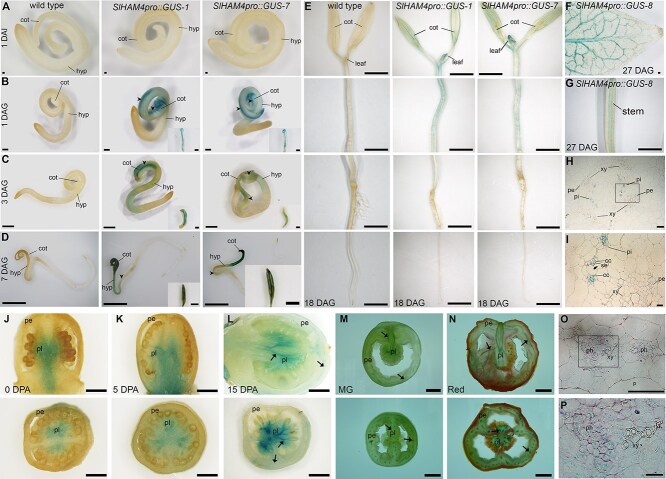
Tissue-specific expression of *SlHAM4*. (**A**–**O**) Histochemical staining for GUS activity in M82 (wild type) and transgenic tomato plants expressing *GUS* driven by *SlHAM4* native promoter (*SlHAM4pro::GUS*). GUS activity was visualized using the chromogenic substrate X-Gluc after ethanol clearing. (**A**) Mature embryos extracted from seeds 1 DAI. (**B**–**E**) Representative whole seedlings at indicated DAG. Insets in **B**–**D** show isolated cotyledons (adaxial side). (**F**) Adaxial side of the first leaf distal half. (**G**) Seedling stem. Scale bars = 200 μm (**A** and **F**), 500 μm (**B**), 1 mm (**C**), 2 mm (**D**, **E,** and **G**). Inset scale bars = 500 μm (**B** and **C**), 1 mm (**D**). cot, cotyledon; hyp, hypocotyl. (**J**–**O**) Manual longitudinal (top panel) and cross (bottom panel) sections of anthesis ovary (**J**) and fruits at indicated developmental stage (**K**–**N**). DPA, days post-anthesis. Arrows indicate GUS staining in representative vasculature. Scale bars 1 mm (**J**–**L**), 1 cm (**M**–**O**). (**H**, **I**, **O,** and **P**) Histological cross-sections of the *SlHAM4pro::GUS-8* tissues stained with Ruthenium red. (**H**) Seedling stem shown in (**G**). (**O**) Pericarp vasculature of mature green fruit shown in (**M**). Scale bar = 100 μm. (**I** and **P**) Higher magnification of the histological sections outlined in (**H**) and (**O**) by a black box. Scale bar = 20 μm. p, parenchyma; ph, phloem; pi, internal phloem; pe, external phloem; xy, xylem; se, sieve element cell; cc, companion cell.

To investigate the spatial distribution of *SlHAM4* in detail, we analyzed its promoter::β-glucuronidase (GUS) activity in transgenic M82 tomato plants. We used the reporter construct *SlHAM4pro::GUS*, which was generated by cloning a 4 kb fragment of the *SlHAM4* promoter upstream of the *GUS* gene (Fig. S4a). This fragment effectively drives native *SlHAM4* expression and restores wild-type phenotypes in *slham4^CRΔ4^* (Fig. 4). Analyzing 12 independent T0 transgenic plants, we observed vasculature-associated GUS staining in the leaves of seven lines (Fig. S4b). These transgenic plants were allowed to set seeds and GUS activity was assayed in their transgenic progeny tissues. GUS staining was not observed in mature embryos extracted from 1 day after imbibition (DAI) transgenic seeds (Fig. 5a). At 1 day after germination (DAG), GUS staining was observed only in the region overlapping the transgenic cotyledons mid-veins (Fig. 5b). This GUS staining pattern was observed also in 3 DAG cotyledons and in addition, at that stage, weak nonspecific GUS staining was observed in the hypocotyl (Fig. 5c). At 7 DAG, stronger GUS staining was observed in the cotyledons mid-vein, as well as in their minor veins. In addition, a weak but specific GUS staining was observed in the vasculature of the hypocotyl (Fig. 5d). At 18 DAG, GUS activity was found throughout the vascular tissues of the cotyledons, leaf primordia, and stem (Fig. 5e). At 27 DAG, developing leaves showed GUS activity in their veins (Fig. 5f) and GUS activity was also observed in the stem vasculature (Fig. 5g). In line with the *SlHAM4* relatively weak expression in M82 seedling roots (Fig. S1a inset), until 27 DAG, GUS staining was not detected in the roots (Fig. 5a–e). Histological sections of 27 DAG *SlHAM4pro::GUS* seedling stem (Fig. 5h and i) and cotyledon major and minor veins (Fig. S5a–d) revealed that GUS staining was confined to the phloem tissue within a vascular bundle. Within the phloem tissue, staining was detected in companion cells (CCs) and sieve elements (SEs) (Fig. 5i, Fig. S5b and c). As the SEs are enucleated [13], the observed SEs’ staining likely reflects transport of the GUS enzyme or movement of its substrate cleavage product from their adjacent CCs. To explore the expression pattern of *SlHAM4* during fruit development, we characterized the GUS staining in ovaries at anthesis and in developing and ripening fruits. Dispersed but specific GUS staining was observed in the placenta and columella of ovaries at anthesis and in very young immature fruits (Fig. 5j, k and Fig. S4c). As the fruits matured and ripened, GUS staining intensity increased and became restricted to the vasculature (Fig. 5l, n and Fig. S4c). These patterns align with the native expression of *SlHAM4* (Fig. S1b–d), supporting its association with vasculature in fruits. Additionally, histology of GUS-stained mature green fruit vascular bundle revealed specific staining in phloem-associated cells (Fig. 5o and p), consistent with its spatial expression in the vascular bundles of seedlings.

### Transcriptomic profiling of *slham4^CRΔ4^* ovaries

**Figure 6:**
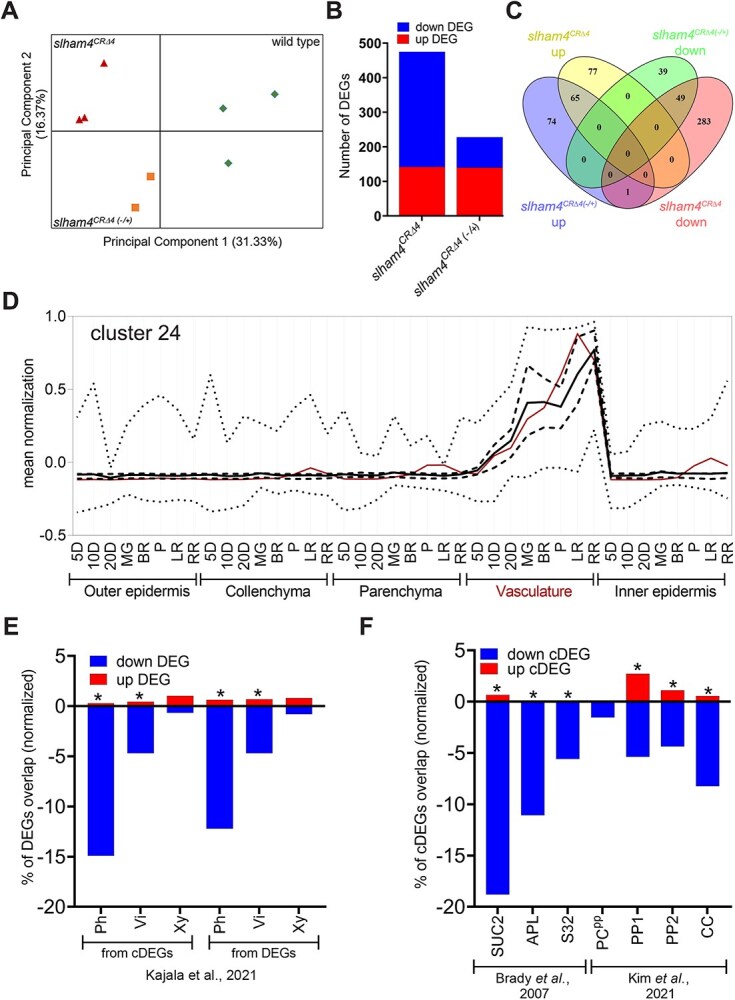
Global gene expression changes in *slham4^CRΔ4^* anthesis ovaries. (**A**) Principal components analysis of all expressed genes showing three distinct groups. (**B**) Total number of DEGs in *slham4^CRΔ4^* mutant and heterozygous (*slham4^CRΔ4(−/+)^*) ovaries compared to wild type. (**C**) Venn diagram displaying specific and overlapping DEGs between *slham4^CRΔ4^* and *slham4^CRΔ4(−/+)^* datasets. (**D**) Expression profile of cluster 24 cDEGs in fruit pericarp tissues based on TEA database data [14]. Cluster-wide average expression is plotted with solid lines, first and third quartiles by dashed lines, and maximum and minimum by dotted lines. The *SlHAM4*-specific profile is plotted with red solid line. (**E** and **F**) Overlap between DEGs and cDEGs (**E**) or Arabidopsis orthologs of cDEGs (**F**) with indicated published vascular datasets. To compensate for dataset size, the numbers of up and down DEGs and cDEGs were normalized relative to the total number of genes in the largest group dataset (Table **S1f**). Asterisks indicate statistical significance of overlap as calculated by http://nemates.org/MA/progs/overlap_stats.cgi. Ph, phloem; Vi, vascular initials; Xy, xylem.

We used RNA sequencing (RNA-seq) to explore the impact of *SlHAM4* absence on the ovary transcriptome and gain insights into the molecular basis underlying tomato fruit catfacing. Comparative transcriptome analysis was conducted on anthesis ovaries from wild-type, heterozygous (*slham4^CRΔ4(−/+)^*), and homozygous (*slham4^CRΔ4^*) M82 plants. For each genotype, three biological replicates of anthesis ovaries were collected for RNA-seq library construction. Each library yielded ~16.5–19.5 million clean sequences, which were mapped to the tomato genome cDNA ITAG 2.5 (Table S1a). Notably, the second biological replicate of the *slham4^CRΔ4(−/+)^* group (*slham4^CRΔ4(−/+)^*-2) showed significant deviation (Fig. S6) and thus was excluded from subsequent analysis. Principal component analysis (PCA) confirmed the reproducibility among biological replicates, highlighting sample differences (Fig. 6a). Applying a fold-change cut-off >2 and a false discovery rate (FDR) <0.01 as significance thresholds, we identified 475 differentially expressed genes (DEGs) in *slham4^CRΔ4^* ovaries compared with wild type. Among these, 142 were upregulated (up) and 333 were downregulated (down) (Fig. 6b and Table S1b). In comparison, the *slham4^CRΔ4(−/+)^* ovaries exhibited 140 up and 88 down DEGs (Fig. 6b and Table S1c). Among identified DEGs, 74 up and 39 down were unique to *slham4^CRΔ4(−/+)^* ovaries, while 77 up and 283 down were unique to *slham4^CRΔ4^* ovaries (Fig. 6c). Given that fruit catfacing was exclusively observed in *slham4^CRΔ4^* fruits, we considered the unique DEGs in their ovaries as potential candidate DEGs (cDEGs) contributing to catfacing. Moreover, 15 overlapping DEGs (1 up and 14 down; Table S1d), exhibiting similar trends and more pronounced fold changes in *slham4^CRΔ4^* compared to *slham4^CRΔ4(−/+)^*, were identified. The differential expression between *slham4^CRΔ4(−/+)^* and *slham4^CRΔ4^* mutants suggests that these DEGs might be regulated by SlHAM4 and could contribute to fruit catfacing. Consequently, these overlapping DEGs were also defined as cDEGs, bringing their total count to 375 (297 down, 78 up; Table S1e).

### The expression of phloem-associated genes is altered in *slham4^CRΔ4^* ovaries

Using gene expression data from the Tomato Expression Atlas (TEA) [14], we performed a cluster analysis to identify cDEGs that are coexpressed with *SlHAM4*, a prerequisite for their regulation by this putative transcriptional regulator. We found that 51 down and 7 up cDEGs co-cluster with *SlHAM4* in cluster 24, which is primarily expressed in the vasculature of developing and ripening fruit pericarp (Fig. 6d and Table S1e). An additional 92 cDEGs exhibited a pericarp vasculature-predominant expression (Fig. S7, clusters 2, 22, 23, 26, 27, 38), bringing the total number of vascular-associated cDEGs to 150, of which 91% (136/150) were downregulated in the *slham4^CRΔ4^* ovaries. To further explore the impact of *SlHAM4* loss-of-function on the transcriptome of ovary vasculature, we compared *slham4^CRΔ4^* DEGs with tomato genes found to be translated in root phloem, vascular initials, or xylem [15] (Table S1f). This comparison revealed that 43% (25/58) of DEGs in cluster 24 are translated in the tomato root phloem, with negligible representation in root vascular initials and xylem (Table S1e). Moreover, 12.8% of the DEGs and 15.2% of the cDEGs were identical to root phloem genes. By contrast, only 3.2% of the DEGs and 1.3% of the cDEGs were identical to root vascular initials and root xylem genes, respectively (Fig. 6e and Table S1e). To identify phloem cell types whose transcriptome was affected due to *slham4* loss-of-function, we first matched each cDEG with its closest Arabidopsis ortholog, then checked for its presence in published Arabidopsis gene lists associated with specific phloem cells in roots [16] and leaves [17]. This bioinformatic approach identified 36 and 35 cDEGs homologous to Arabidopsis root and leaf phloem-associated genes, respectively, mostly downregulated. Notably, the overlap was greatest with CC-specific genes in both root (SUC2, 10/36) and leaf (CC, 26/35) (Fig. 6f). Intriguingly, the leaf CC-specific gene list includes the gene code for HAM4 (AT4G36710), the Arabidopsis homolog of SlHAM4, and the Arabidopsis orthologs of 17.24% (10/58) of cluster 24 genes (Table S1e).

We annotated the tomato orthologs of key Arabidopsis genes involved in phloem development among the downregulated cDEGs. Included are the tomato orthologs of the MYB transcription factor ALTERED PHLOEM DEVELOPMENT (APL; Solyc12g017370), crucial for protophloem SEs differentiation [18]; NAC-DEPENDENT EXONUCLEASE 1 (NEN1; Solyc03g115780), which promotes SE nuclear degradation and is transcriptionally regulated by the APL targets NAC45 and NAC86 [19]; LATERAL ROOT DEVELOPMENT 3 (LRD3; Solyc05g052910), involved in phloem development and function [20]; PHLOEM INTERCALATED WITH XYLEM (PXY; Solyc05g051640), a kinase critical for cambium cell divisions [21]; XYLEM INTERMIXED WITH PHLOEM 1 (XIP1; Solyc04g077010), regulating SE cell morphology [22]; VND7-INTERACTING 2 (VNI2; Solyc03g097650), a NAC transcription factor involved in phloem specification [23]; ATP-BINDING CASSETTE G14 (ABCG14; Solyc08g075430), required for phloem development [24]; and the CC-specific HSP20-like chaperone (AT5G54660/NPCC8, Solyc07g064020) [25].

In addition, we also identified tomato orthologs of Arabidopsis genes essential for phloem functions among downregulated cDEGs. These include the phloem-localized sulfate transporter SULTR1.3 (Solyc12g056930) [26]; Phloem Protein 2 (PP2) and related lectins (Solyc02g069060, Solyc02g069020, Solyc02g069030, Solyc03g121300, Solyc00g048510, Solyc10g078600) that are expressed in CCs and thought to be involved in the long-distance movement of RNAs and defense [27]; five orthologs of Thioredoxin, including three of Thioredoxin h (TRXh) (Solyc05g006830, Solyc05g006850, Solyc05g006870), which has been identified as a major protein in the phloem exudates of various monocots and dicots [28–30] and was detected in rice leaf companion cells [31]; CLAVATA1 (Solyc04g081590), which was shown to be expressed in root and leaf CCs and regulates lateral root outgrowth under N-deficient conditions [16, 32]; two orthologs of MYB-RELATED PROTEIN 2 (MYR2, Solyc10g085620, Solyc10g083340), linked to nitrogen uptake and assimilation; FT-INTERACTING PROTEIN 1 (Solyc03g077920), interacting with FLOWERING LOCUS T (FT) in the CCs to mediate its phloem export into SEs [33]; EARLY FLOWERING MYB PROTEIN (EFM; Solyc01g108300), a root- and leaf CCs-expressed MYB transcription factor that is involved in negative regulation of flowering [34]; and a tomato ortholog of SODIUM POTASSIUM ROOT DEFECTIVE 1 (NaKR1; Solyc10g085910), a phloem metal binding protein that is expressed in CCs and necessary for phloem function including the long-distance movement of FT [35].

## Discussion

This study presents a comprehensive functional analysis of *SlHAM4* in tomato, revealing its requirement for development and phloem function. Our findings suggest that SlHAM4 is involved in gene regulation within the phloem system.

Public expression data and GUS reporter assays demonstrated that *SlHAM4* is predominantly expressed in the vasculature of various organs. The plant vasculature is composed of xylem, which consists of nonliving cells and conducts water and minerals, and phloem, which is composed of living cells and transports essential nutrients like sugars, amino acids, and hormones [36]. Histochemical GUS staining revealed that within the vascular bundle, *SlHAM4* expression is confined to phloem tissues, contrasting with an absence of expression in the nearby xylem. This phloem-specific expression is supported by the detection of *SlHAM4* mRNA within the phloem translatome, the set of actively translated mRNAs, in tomato seedling roots [15], and aligns with the expression of Arabidopsis *HAM4/SCL15* detected by GUS staining in *SCL15pro::GUS* seedlings [7].

The abundance of *SlHAM4* in mature organs, such as ripening fruits, and the lack of obvious developmental defects in the phloem of *slham4^CRΔ4^* suggest that SlHAM4 is involved in phloem functionality rather than development. This role may be conserved in Arabidopsis, given the nearly wild-type phenotype of the *ham4/scl15* mutant and the expression of *HAM4* in mature roots [7]. In Arabidopsis, HAM4 interacts with HDA19 and WOX4, affecting gene expression [3, 7]. In our study, the absence of *SlHAM4* modified the transcriptome of anthesis ovaries, including the downregulation of phloem-associated genes. This raises the possibility that SlHAM4 may act as a transcriptional regulator by interacting with as-yet unidentified phloem proteins.

The *Arabidopsis pxy* and *xip1* mutants exhibit impaired phloem organization, which leads to disrupted phloem function and is associated with anthocyanin overaccumulation in cotyledons and leaves [21, 22]. Similarly, the *slham4^CR^* mutants show anthocyanin overaccumulation in vegetative tissues, along with reduced expression of *XIP1* and *PXY*, supporting the notion that phloem function is compromised in these mutants. Phloem tissue primarily comprises three main cell types: the enucleate SEs that form the sieve tube, the adjacent CCs, which support the SEs through connecting plasmodesmata, and phloem parenchyma cells [36]. Within the phloem tissue, GUS staining detected the expression of *SlHAM4* in phloem-associated cells including CCs. These results are consistent with observations in Arabidopsis, where the SCL15/HAM4-GFP fusion protein was localized to phloem-specialized cells, including CCs [7], and single-cell transcriptomics identified *HAM4* mRNA specifically in the CCs of leaves [17]. Moreover, our transcriptomic analysis revealed that the absence of SlHAM4 activity leads to reduced expression of several genes known to be expressed in CCs. This may influence the protein composition of the phloem, thereby affecting its functionality. A significant reduction in the expression of *TRXh* genes, known for their roles in maintaining the proper function of various target proteins by ensuring correct disulfide bonding [37], in *slham4^CRΔ4^*, exemplifies the potential impact of SlHAM4 absence on phloem protein content. Members of the *TRXh* family are known for their expression in CCs and abundance in the phloem [38]. The decrease in TRXh levels within the phloem could compromise target protein functionality by allowing the oxidation of redox-sensitive cysteines. These findings collectively suggest a broader regulatory role for SlHAM4 in various aspects of phloem function.

In tomato, phloem-mediated transport of photosynthates and essential nutrients is vital for meeting the metabolic requirements of the developing fruit, a major sink organ, thereby supporting its growth [39]. We observed a marked size reduction in *slham4^CR^* fruits compared to those of the M82 cultivar, indicating inhibited growth of mutant fruits. This and the specific *SlHAM4* expression in the phloem of developing fruits raise the possibility that compromised phloem function in *slham4^CR^* mutant fruits may impede the transport of photosynthates and essential nutrients, adversely affecting their normal growth. Furthermore, our study revealed that the absence of functional *SlHAM4* led to small ruptures in the pericarp of mutant setting fruits, which ultimately caused significant scarring in the mature fruits. This phenotype closely resembles the catface syndrome in tomatoes, a disorder characterized by large, irregular scars on the fruit's blossom end [10, 40]. Previous study suggested that fruit catfacing originates from the incomplete closure of carpels at the base of the style in rapidly growing fertilized ovaries. This defect may result from the overproliferation of ovary tissues, particularly the locules and style, during flower development. Accordingly, brief cold stress or gibberellin treatment during flowering promoted ovary tissue proliferation such as style fasciation and increased locule number, which is associated with increased fruit catfacing [41]. Recent study has demonstrated a link between cold stress and the disruption of the WUS-CLV3 feedback loop, which led to stem cell proliferation in the tomato flower meristem [42]. This disruption provides a possible explanation for cold-induced tomato fruit catfacing. However, the *slham4^CR^* mutant flowers display normal-looking pistils at anthesis, indicating that catfacing in these mutants is not due to overproliferation of ovary tissues. Notably, in certain cold-treated tomato varieties, fruit catfacing was not associated with ovary tissue proliferation. It was suggested that cold temperatures might inhibit the formation or transport of growth substances critical for normal cell division near the style's base, preventing complete carpel closure [11]. This raises the possibility that suboptimal phloem function in *slham4^CRΔ4^* anthesis ovaries failed to support the rapid growth of the setting fruit pericarp leading to the formation of small ruptures that later expand into scars, although the specific cause for scar formation at particular locations remains unclear.

In summary, our results suggest that tomato *HAM4* is a phloem-associated gene that is necessary for proper development, as well as for phloem integrity, in part by modulating the expression of phloem genes, particularly those expressed in CCs. Further research is needed to identify which CC genes are transcriptionally regulated by SlHAM4 and to clarify their specific roles in development related to phloem function.

## Materials and methods

### Plant material and growth conditions

Germinated tomato cv. M82 seedlings and regenerated T0 plants were grown in a growth chamber at 24^ο^C under 50–70 μmol m^−2^ s^−1^ photosynthetic photon flux density with 16/8-h light/dark period. Subsequently, 1-month-old seedlings and acclimated regenerated plants were transplanted into 4 l pots containing a tuff-peat mix soil. These plants were grown under greenhouse conditions, with temperatures maintained between 25°C and 30°C (day/night). Crosses were made in emasculated flowers by hand pollination.

### Plasmids construction

For pBI101-SlHAM4pro::GUS binary plasmid, a 4000 bp sequence upstream of the *SlHAM4* start codon was polymerase chain reaction (PCR)-amplified from the M82 tomato genomic DNA using the primer pair SlHAM4pro-BamHI-F1 and SlHAM4pro-XmaI-R that contained *BamH*I- and *Sma*I-flanking restriction sites. Following *BamH*I and *Sma*I digestion, the amplified fragment was cloned into the corresponding sites of the binary plasmid pBI101 just upstream of *GUS*. For the pART27-35S::SlHAM4 binary plasmid, a 1611 bp encoding *SlHAM4* was PCR-amplified from an anthesis ovary cDNA with the primer pair SlHAM4-SalI-Fwd and SlHAM4-HindIII-Rev (primer sequences can be found in Table S2). The amplified fragment was digested with *Sal*I and *Hind*III and subsequently cloned into *Xho*I and *Hind*III sites of the pART27-35S binary vector downstream of the *35S* promoter. The pDGB3-Omega1-SlHAM4pro::SlHAM4-GFP::TNOS binary plasmid was constructed using the GoldenBraid cloning system. *GFP* (with Gly-Gly-Ser linker) was PCR-amplified from the *AtSUC2pro*::*GFP* tomato line genomic DNA [43]. The 4 kb SlHAM4pro and *GFP* PCR amplicons were individually cloned into the GoldenBraid entry vector (level 0), pUPD2 [44]. The expression cassette was precisely generated in level 1 binary plasmid pDGB3-Alpha2 by assembling *SlHAM4pro*, *SlHAM4*, and *GFP* with additional *NOS* terminator (*TNOS*). The constructed expression cassette was further integrated with plant kanamycin resistance cassette pNOS::nptII::TNOS in the level 2 binary plasmid pDGB3-Omega1 to generate the final binary plasmid. All binary constructs were validated by sequencing. Primer sequences used for plasmid constructions are listed in Table S2.

### CRISPR/Cas9-mediated mutagenesis of *SlHAM4*

First, two gene-specific guide RNAs (gRNAs) targeting the coding region of *SlHAM4* were designed (gRNA sequences are provided in Table S2) and each was incorporated *in silico* into sgRNA. Then, a construct containing the two sgRNAs in tandem each driven by the synthetic Arabidopsis U6 promoter delimited by 5’-*Mlu*I and 3’-*Hind*III was artificially synthesized (GeneWiz, USA) and cloned into pUC57 to generate pUC57-U6::sgRNA1-U6::sgRNA2. The pUC57-U6::sgRNA1-U6::sgRNA2 plasmid was digested with *Mlu*I and *Hind*III restriction enzymes, and the released U6::sgRNA1-U6::sgRNA2 fragment was ligated into the corresponding sites of pRCS binary vector, which also contained a plant codon-optimized version of Cas9 driven by the 35S promoter [45]. To detect the CRISPR/Cas9-induced mutations in *SlHAM4*, the genomic DNA was extracted from each transgenic T0 plant and screened by PCR for the presence of the *35S::Cas9* transgene with the primer pair Cas9-Fwd and Cas9-Rev. Then, the *SlHAM4*-targeted sequences in the transgenic T0 plants were PCR-amplified with primer pair SlHAM4-Cas9-valid-F and SlHAM4-Cas9valid-R and sequenced to identify indels in them. The T0 plants carrying mutation were backcrossed to M82 wild type, and the resulting F1 progeny plants were genotyped for the absence of the *35S::Cas9* transgene and presence of indels. The identified nontransgenic F1 CRISPR mutants were selfed, and homozygous F2 CRISPR mutants were genotyped by sequencing. Detection of homozygous *slham4^CRΔ4^* and *slham4^CRΔ3^* mutants was further done by PCR with the primer pair CR-SlHAM4delta4detect-fwd and SlHAM4-Cas9 valid-R, which amplify only the *SlHAM4* wild-type allele. Primer sequences are listed in Table S2.

### Tomato transformations

Binary constructs were transformed into tomato cv. M82 (pBI101-SlHAM4pro::GUS, pRCS-2xU6syn::SlHAM4, pART27-35S::SlHAM4) or *slham4^CRΔ4^* (pDGB3-Omega1-SlHAM4pro::SlHAM4-GFP) plants by cocultivation of 12-day-old cotyledons with *Agrobacterium* strain GV3101 as described previously [46].

### Histochemical GUS staining

For GUS staining, *SlHAM4pro::GUS* tomato transgenic tissues were submerged in GUS staining buffer [100 mM sodium phosphate buffer (pH 7.0), 2 mM potassium ferricyanide, 2 mM potassium ferrocyanide, 0.1% Triton X-100, 10 mM EDTA and 1 mM X-Gluc] followed by 1 h of vacuum infiltration with six intermittent stops and overnight incubation in 37°C. Three washes with 70% ethanol (v/v) followed by incubation at 37°C for 8 h after each wash dechlorophylized the stained tissues, which were then visualized or used for histology.

### Histology

GUS-stained and unstained tissues were fixed in paraformaldehyde (PFA) solution as described in Hendelman et al., (2016). Microtome-cut sections of 4-μm thick were mounted on microscopic slides and stained with 0.1% (w/v) Toluidine blue for 1 min (flower pedicel and leaf rachis) or 0.1% (w/v) Safranin for 1 h followed by 0.1% (w/v) Fast Green for 30 s (ovary and fruit). For GUS-stained tissues microtome-cut sections of 10-μm thick were mounted on microscopic slides and further stained with 0.05% (w/v) Ruthenium red for 1 min. Slides were examined and photographed under bright field using an Olympus DP73 microscope equipped with a digital camera.

### Transcriptome analysis by RNA-seq

Total RNA was extracted from isolated ovaries using Bio-Tri RNA reagent (Bio-Lab, Israel) as described by the manufacturer's protocol. For RNA-seq, three biological replicates of wild-type, *slham4^CRΔ4^*, and *slham4^CRΔ4(−/+)^* isolated anthesis ovaries were used. Each replicate contained 10 ovaries from eight independent plants. RNA-seq libraries (Illumina Truseq RNA) preparation and pair-end sequencing were performed at MACROGEN-EUROPE (Macrogen, The Netherlands). Differential expression analysis was done at the ARO bioinformatic unit. Briefly, raw reads underwent a filtering and cleaning procedure. The SortMeRNA tool was used to remove rRNA sequences. The FASTX Toolkit (version 0.0.13.2) was then employed to trim read-end nucleotides with quality scores <30 using the FASTQ Quality Trimmer, and to discard reads with <70% base pairs having a quality score of ≥30 using the FASTQ Quality Filter. Reads were mapped to the tomato coding sequences (ITAG2.4 release provided by the International Tomato Annotation Group) using Bowtie2. Transcript quantification was performed with the Expectation–Maximization method (RSEM), utilizing the align_and_estimate_abundance.pl script from the Trinity software package (https://github.com/trinityrnaseq/trinityrnaseq/wiki). PCA was conducted using R Bioconductor. Differential expression analysis was carried out with the edgeR R package, considering genes with an FDR <0.01 and at least a 2-fold change as differentially expressed.

### Quantitative PCR analyses

Total RNA was extracted from indicated tissue using Bio-Tri RNA reagent (Bio-Lab) as described by the manufacturer's protocol. Two micrograms of total RNA were treated with DNase I, followed by 40 cycles of PCR to ensure the absence of genomic DNA in the samples. First-strand cDNA was then synthesized from 1 μg of total RNA using a Maxima first-strand cDNA synthesis kit (Thermo Scientific, Lithuania) according to the manufacturer's instructions. Quantitative PCR (qPCR) was performed on a StepOnePlus system (Thermo Scientific), and the results were analyzed with StepOne software version 2.2.2 (Thermo Scientific). Relative expression levels were normalized using *SlTIP41* as a reference gene and calculated using the comparative delta delta Ct (∆∆Ct) method. The primers for qPCR are listed in Table S2.

### Bioinformatic analysis

The TEA database (http://tea.solgenomics.net/) gene expression levels (average RPM) of *SlHAM4* and indicated DEGs in the fruit pericarp cell types across stages (5D to RR) were used for K-means clustering, which was done using Morpheus website (https://software.broadinstituteorg/morpheus) with number of row clusters = 40. The mean normalized value for each DEG per sample (fruit pericarp cell type at a specific stage) was calculated using the formula (X − X_av_) / (X_max_ − X_min_), where X is the average RPM value of a specific DEG, and X_av_, X_min_, and X_max_ are the mean, minimum, and maximum average RPM values of that DEG across all samples, respectively. The Arabidopsis orthologs of identified tomato DEGs were determined using the Best-Hits-and-Inparalogs (BHIF) method at https://bioinformatics.psb.ugent.be/plaza/versions/plaza_v4_5_dicots/download.

### Statistical analyses

Statistical analyses were conducted using GraphPad Prism 8.3, unless stated otherwise.

## Supplementary Material

Web_Material_uhae325

## Data Availability

The RNA-seq data is available from the SRA database under the accession number PRJNA682014. The candidate DEGs and *SlHAM4* expression profiles in pericarp cell types tissue across the stages (5DPA to RR) of tomato fruit were retrieved from the Tomato Expression Atlas database (http://tea.solgenomics.net/) [47].

## References

[1] Stuurman J, Jäggi F, Kuhlemeier C. Shoot meristem maintenance is controlled by a *GRAS*-gene mediated signal from differentiating cells. Genes Dev. 2002;16:2213–8 12208843 10.1101/gad.230702 PMC186665

[2] Engstrom EM, Andersen CM, Gumulak-Smith J. et al. Arabidopsis homologs of the petunia *HAIRY MERISTEM* gene are required for maintenance of shoot and root indeterminacy. Plant Physiol. 2010;155:735–50 21173022 10.1104/pp.110.168757 PMC3032463

[3] Zhou Y, Liu X, Engstrom EM. et al. Control of plant stem cell function by conserved interacting transcriptional regulators. Nature. 2015;517:377–80 25363783 10.1038/nature13853 PMC4297503

[4] Llave C, Xie Z, Kasschau KD. et al. Cleavage of *Scarecrow-like* mRNA targets directed by a class of Arabidopsis miRNA. Science. 2002;297:2053–6 12242443 10.1126/science.1076311

[5] Han H, Geng Y, Guo L. et al. The overlapping and distinct roles of *HAM* family genes in Arabidopsis shoot meristems. Front Plant Sci. 2020;11:1375 10.3389/fpls.2020.541968 PMC7498855 33013964

[6] Geng Y, Zhou Y. *HAM* gene family and shoot meristem development. Front Plant Sci. 2021;12:800332 34987539 10.3389/fpls.2021.800332 PMC8720772

[7] Gao M-J, Li X, Huang J. et al. SCARECROW-LIKE15 interacts with HISTONE DEACETYLASE19 and is essential for repressing the seed maturation programme. Nat Commun. 2015;6:7243 26129778 10.1038/ncomms8243 PMC4507008

[8] Hendelman A, Kravchik M, Stav R. et al. Tomato *HAIRY MERISTEM* genes are involved in meristem maintenance and compound leaf morphogenesis. J Exp Bot. 2016;67:6187–200 27811085 10.1093/jxb/erw388 PMC5100029

[9] Bolle C . The role of GRAS proteins in plant signal transduction and development. Planta. 2004;218:683–92 14760535 10.1007/s00425-004-1203-z

[10] Peet MM . Physiological disorders in tomato fruit development. Acta Hortic. 2009;821:151–60

[11] Knavel DE, Mohr HC. Some abnormalities in tomato fruits as influenced by cold treatment of seedlings. J Am Soc Hortic Sci. 1969;94:411–3

[12] Gillaspy G, Ben-David H, Gruissem W. Fruits: a developmental perspective. Plant Cell. 1993;5:1439–51 12271039 10.1105/tpc.5.10.1439 PMC160374

[13] Imlau A, Truernit E, Sauer N. Cell-to-cell and long-distance trafficking of the green fluorescent protein in the phloem and symplastic unloading of the protein into sink tissues. Plant Cell. 1999;11:309–22 10072393 10.1105/tpc.11.3.309 PMC144181

[14] Fernandez-Pozo N, Zheng Y, Snyder SI. et al. The tomato expression atlas. Bioinformatics. 2017;33:2397–8 28379331 10.1093/bioinformatics/btx190 PMC5860121

[15] Kajala K, Gouran M, Shaar-Moshe L. et al. Innovation, conservation, and repurposing of gene function in root cell type development. Cell. 2021;184:3333–3348.e19 34010619 10.1016/j.cell.2021.04.024

[16] Brady SM, Orlando DA, Lee JY. et al. A high-resolution root spatiotemporal map reveals dominant expression patterns. Science. 2007;318:801–6 17975066 10.1126/science.1146265

[17] Kim JY, Symeonidi E, Pang TY. et al. Distinct identities of leaf phloem cells revealed by single cell transcriptomics. Plant Cell. 2021;33:511–30 33955487 10.1093/plcell/koaa060 PMC8136902

[18] Bonke M, Thitamadee S, Mähönen AP. et al. APL regulates vascular tissue identity in Arabidopsis. Nature. 2003;426:181–6 14614507 10.1038/nature02100

[19] Furuta KM, Yadav SR, Lehesranta S. et al. Arabidopsis NAC45/86 direct sieve element morphogenesis culminating in enucleation. Science. 2014;345:933–7 25081480 10.1126/science.1253736

[20] Ingram P, Dettmer J, Helariutta Y. et al. Arabidopsis *lateral root development 3* is essential for early phloem development and function, and hence for normal root system development. Plant J. 2011;68:455–67 21749503 10.1111/j.1365-313X.2011.04700.x

[21] Fisher K, Turner S. PXY, a receptor-like kinase essential for maintaining polarity during plant vascular-tissue development. Curr Biol. 2007;17:1061–6 17570668 10.1016/j.cub.2007.05.049

[22] Tabata R, Sumida K, Yoshii T. et al. Perception of root-derived peptides by shoot LRR-RKs mediates systemic N-demand signaling. Science. 2014;346:343–6 25324386 10.1126/science.1257800

[23] Yamaguchi M, Ohtani M, Mitsuda N. et al. VND-INTERACTING2, a NAC domain transcription factor, negatively regulates xylem vessel formation in Arabidopsis. Plant Cell. 2010;22:1249–63 20388856 10.1105/tpc.108.064048 PMC2879754

[24] Le Hir R, Sorin C, Chakraborti D. et al. ABCG9, ABCG11 and ABCG14 ABC transporters are required for vascular development in Arabidopsis. Plant J. 2013;76:811–24 24112720 10.1111/tpj.12334

[25] Zhang C, Barthelson RA, Lambert GM. et al. Global characterization of cell-specific gene expression through fluorescence-activated sorting of nuclei. Plant Physiol. 2008;147:30–40 18354040 10.1104/pp.107.115246 PMC2330299

[26] Yoshimoto N, Inoue E, Saito K. et al. Phloem-localizing sulfate transporter, Sultr1;3, mediates re-distribution of sulfur from source to sink organs in Arabidopsis. Plant Physiol. 2003;131:1511–7 12692311 10.1104/pp.014712 PMC166910

[27] Pallas V, Gómez G. Phloem RNA-binding proteins as potential components of the long-distance RNA transport system. Front Plant Sci. 2013;4:130 23675378 10.3389/fpls.2013.00130 PMC3650515

[28] Ishiwatari Y, Honda C, Kawashima I. et al. Thioredoxin h is one of the major proteins in rice phloem sap. Planta. 1995;195:456–63 7766047 10.1007/BF00202605

[29] Schobert C, Baker L, Szederkényi J. et al. Identification of immunologically related proteins in sieve-tube exudate collected from monocotyledonous and dicotyledonous plants. Planta. 1998;206:245–52

[30] Carella P, Merl-Pham J, Wilson DC. et al. Comparative proteomics analysis of phloem exudates collected during the induction of systemic acquired resistance. Plant Physiol. 2016;171:1495–510 27208255 10.1104/pp.16.00269 PMC4902610

[31] Ishiwatari Y, Nemoto K, Fujiwara T. et al. In situ hybridization study of the rice phloem thioredoxin h mRNA accumulation – possible involvement in the differentiation of vascular tissues. Physiol Plant. 2000;109:90–6

[32] Araya T, Miyamoto M, Wibowo J. et al. CLE-CLAVATA1 peptide-receptor signaling module regulates the expansion of plant root systems in a nitrogen-dependent manner. Proc Natl Acad Sci. 2014;111:2029–34 24449877 10.1073/pnas.1319953111 PMC3918772

[33] Liu L, Liu C, Hou X. et al. FTIP1 is an essential regulator required for florigen transport. PLoS Biol. 2012;10:e1001313 22529749 10.1371/journal.pbio.1001313 PMC3328448

[34] Yan Y, Shen L, Chen Y. et al. A MYB-domain protein EFM mediates flowering responses to environmental cues in Arabidopsis. Dev Cell. 2014;30:437–48 25132385 10.1016/j.devcel.2014.07.004

[35] Zhu Y, Liu L, Shen L. et al. NaKR1 regulates long-distance movement of FLOWERING LOCUS T in Arabidopsis. Nat Plants. 2016;2:1–10 10.1038/nplants.2016.75 27255839

[36] Hardtke CS . Phloem development. New Phytol. 2023;239:852–67 37243530 10.1111/nph.19003

[37] Gelhaye E, Rouhier N, Jacquot JP. The thioredoxin h system of higher plants. Plant Physiol Biochem. 2004;42:265–71 15120110 10.1016/j.plaphy.2004.03.002

[38] Reichheld JP, Mestres-Ortega D, Laloi C. et al. The multigenic family of *thioredoxin h* in Arabidopsis thaliana: specific expression and stress response. Plant Physiol Biochem. 2002;40:685–90

[39] Ho LC . The mechanism of assimilate partitioning and carbohydrate compartmentation in fruit in relation to the quality and yield of tomato. J Exp Bot. 1996;47:1239–43 21245255 10.1093/jxb/47.Special_Issue.1239

[40] Srinivasulu B, Rao GS, Singh DPK. Physiological disorders of tomato and their management. J Pharmacogn Phytochem. 2020;9:2149–50

[41] Sawhney VK . The role of temperature and its relationship with gibberellic acid in the development of floral organs of tomato (*Lycopersicon esculentum*). Can J Bot. 1982;61:1258–65

[42] Wu J, Sun W, Sun C. et al. Cold stress induces malformed tomato fruits by breaking the feedback loops of stem cell regulation in floral meristem. New Phytol. 2022;237:2268–83 10.1111/nph.18699 36564973

[43] Spiegelman Z, Ham BK, Zhang Z. et al. A tomato phloem-mobile protein regulates the shoot-to-root ratio by mediating the auxin response in distant organs. Plant J. 2015;83:853–63 26173789 10.1111/tpj.12932

[44] Sarrion-Perdigones A, Falconi EE, Zandalinas SI. et al. GoldenBraid: an iterative cloning system for standardized assembly of reusable genetic modules. PLoS One. 2011;6:e21622 21750718 10.1371/journal.pone.0021622 PMC3131274

[45] Damodharan S, Corem S, Gupta SK. et al. Tuning of *SlARF10A* dosage by sly-miR160a is critical for auxin-mediated compound leaf and flower development. Plant J. 2018;96:855–68 30144341 10.1111/tpj.14073

[46] Damodharan S, Zhao D, Arazi T. A common miRNA160-based mechanism regulates ovary patterning, floral organ abscission and lamina outgrowth in tomato. Plant J. 2016;86:458–71 26800988 10.1111/tpj.13127

[47] Shinozaki Y, Nicolas P, Fernandez-Pozo N. et al. High-resolution spatiotemporal transcriptome mapping of tomato fruit development and ripening. Nat Commun. 2018;9:364 29371663 10.1038/s41467-017-02782-9 PMC5785480

