# Supplementary material for: Tomato HAIRY MERISTEM4, expressed in the phloem, is required for proper shoot and fruit development
Source: Hortic Res. 2024 Nov 21;12(3):uhae325. doi: 10.1093/hr/uhae325 (PMC11879553; doi:10.1093/hr/uhae325)
Supplement: Web_Material_uhae325 [file web_material_uhae325.zip › Khedia et al Supplemental Data_190924.pdf]

Supplementary information

Tomato *HAIRY MERISTEM 4* is required for proper shoot and fruit development

Jackson Khedia[1], Abhay Pratap Vishwakarma[2], Ortal Galsurker[1], Shira Corem[3], Suresh Kumar Gupta[4], Tzahi Arazi[1*]

[1]Institute of Plant Sciences, Agricultural Research Organization, Volcani Center, 68 HaMaccabim Road, P.O.B 15159 Rishon LeZion 7505101, Israel.
[2]Current address: Department of Botany, Deshbandhu College, University of Delhi, New Delhi, India.
[3]Current address: BetterSeeds Ltd. Birkat Am 54, Givat Hen 43905, POB 46, Israel.
[4]Current address: Department of Biochemistry and Molecular Biology, Michigan State University, East Lansing, MI 48824, USA.

*To whom correspondence should be addressed: Email: tarazi@agri.gov.il

Tzahi Arazi, Tel: +972-3-9683498; Email: tarazi@agri.gov.il
Institute of Plant Sciences, ARO, Volcani Center, HaMaccabbim Road 68, Rishon LeZion 7505101, Israel.

The following Supplemental information is available for this article:

Table S1. RNA-seq mapping statistics and lists of DEGs between stage 18 ovaries of wild type, *slham4*$^{CR\Delta4(-/+)}$ and *slham4*$^{CR\Delta4}$.
Table S2. Primers and gRNAs used in this study.

Supplementary figures

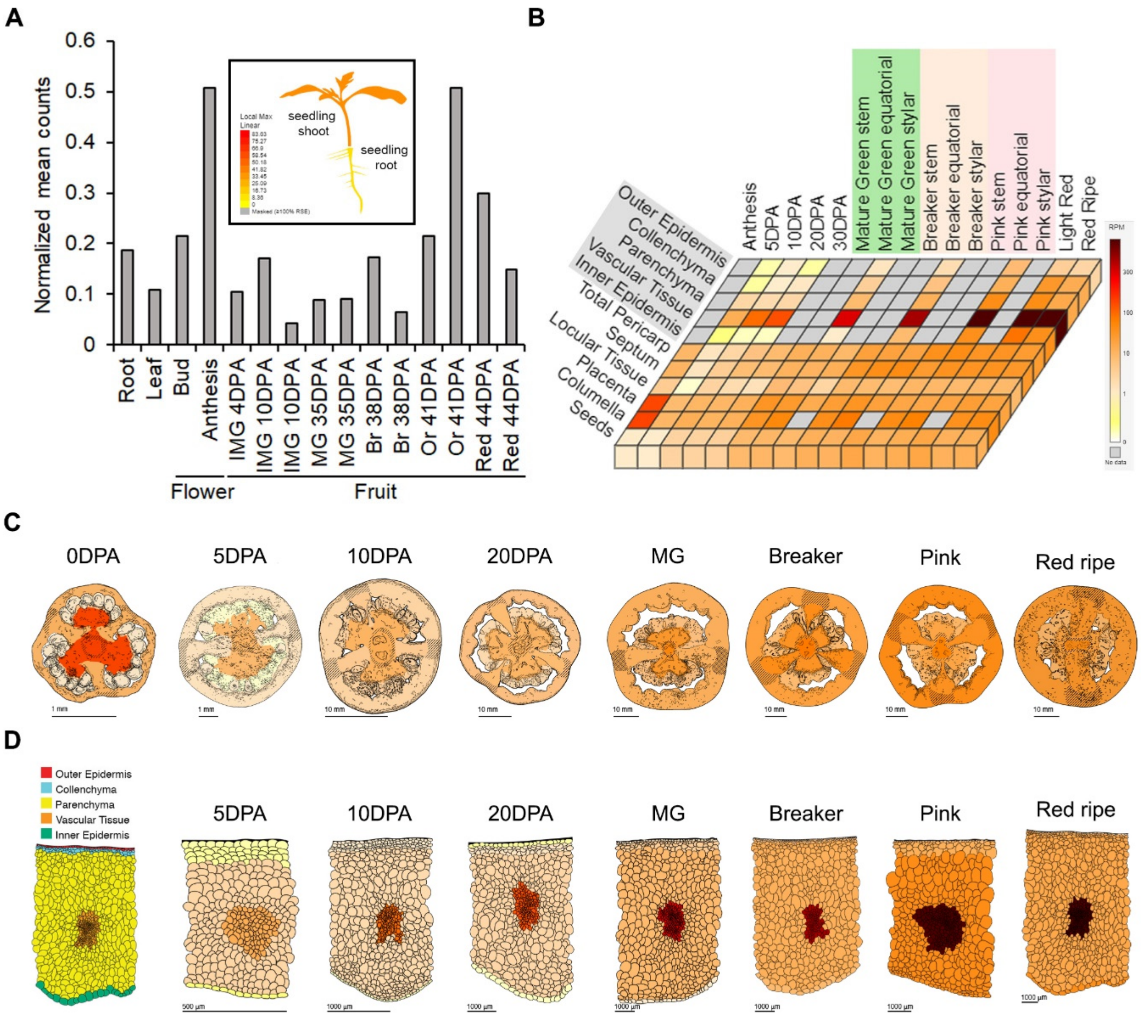

Figure S1. Expression patterns of *SlHAM4* according to public databases. (**A**) Transcript levels of *SlHAM4* in various tissues of tomato cv. Alisa Craig and seedling tissues of tomato cv. M82 (inset). Values represent normalized mean counts. Data for Alisa Craig were sourced from the TomExpress database [1], and for M82 from ePLANT [2]. IMG, immature green; MG, mature green; Br, breaker; Or, orange. (**B** to **D**) Tissue based expression profiles of *SlHAM4*. (**B**) expression cube; (**C**) fruits equatorial regions; (**D**) laser dissected fruit pericarps. Days post anthesis (DPA) are indicated. Expression levels are depicted in colors corresponding to Reads Per Million (RPM) values, as outlined in **B**. Data source is the Tomato Expression Atlas (TEA) [3].

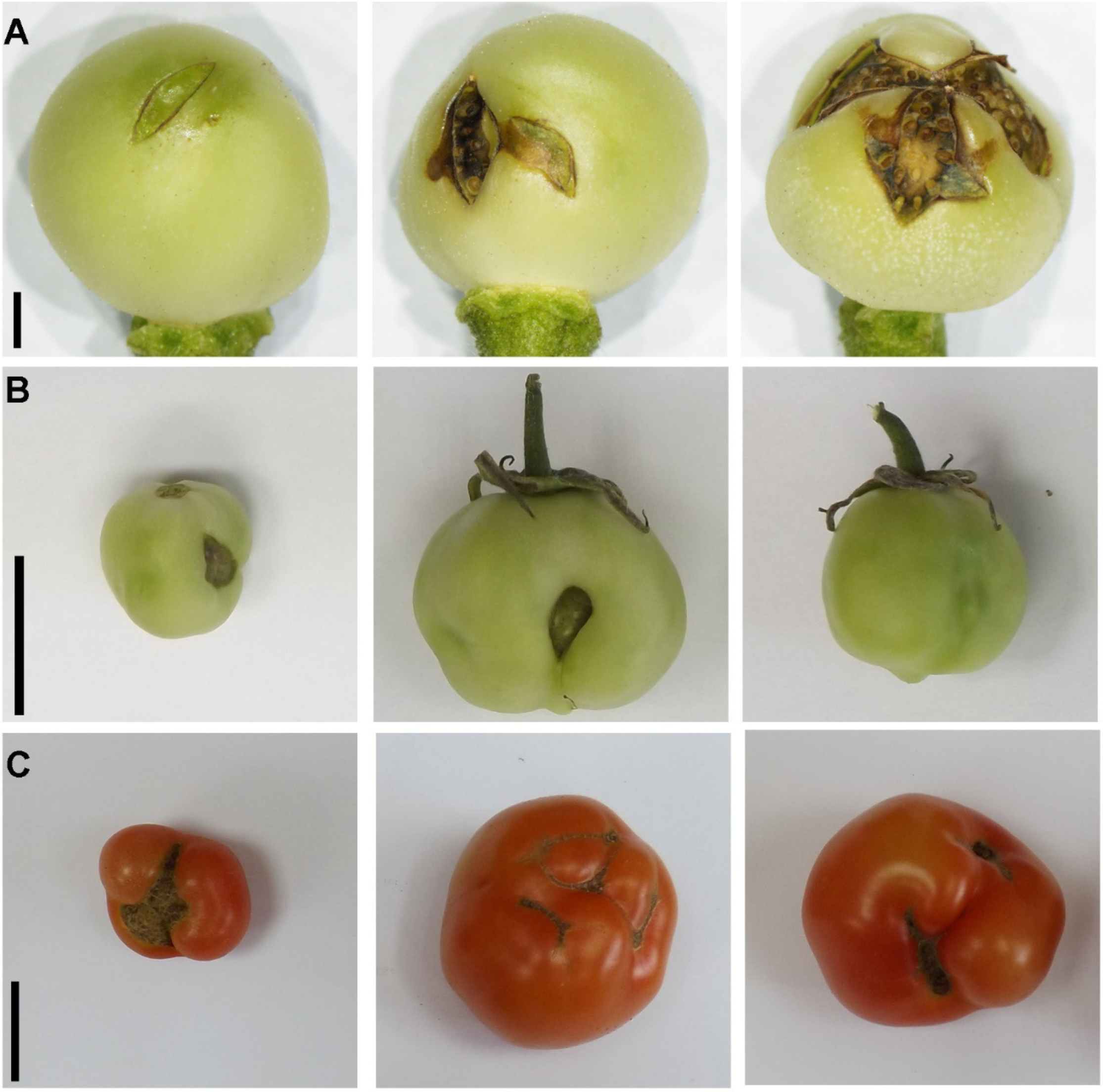

Figure S2. Variation of the catface phenotype in *slham4*$^{CR\Delta 4}$ fruits. (**A**) 11 mm immature green fruits. Scale bars = 2 mm. (**B**) 1-2 cm immature green fruits. Scale bars = 2 cm. (**C**) Red ripe fruits. Scale bars = 2 cm.

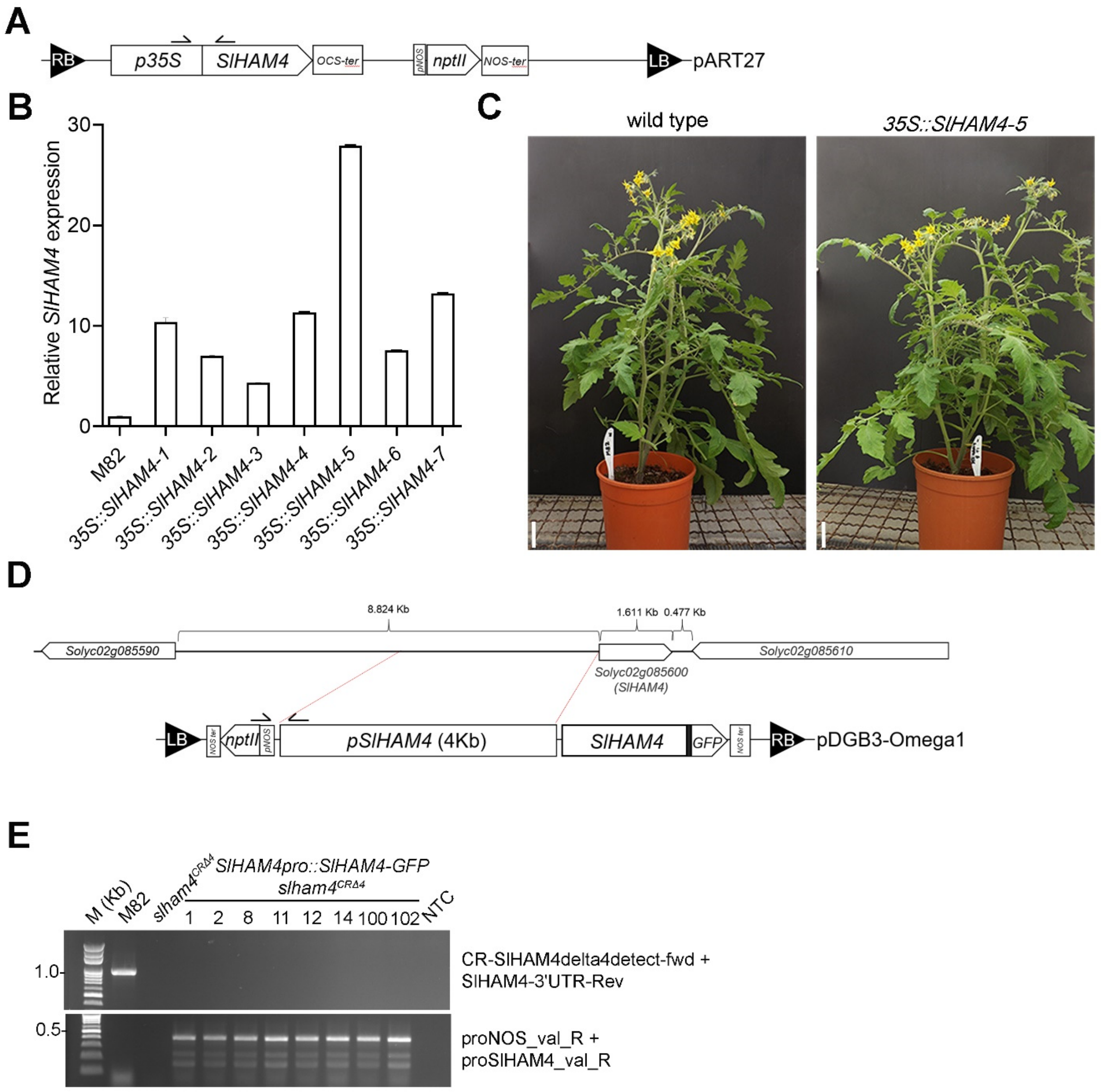

Figure S3. Construction of complementation constructs and validation of transgenic plants. (**A**, **D**) Schematic of the binary constructs introduced into M82 (**A**) and *slham4*$^{CR\Delta4}$ (**D**). *SlHAM4* indicates *SlHAM4* coding sequence. The Gly-Gly-Ser linker between SlHAM4 and GFP is indicated by a black rectangle. The position of primers used for transgene validation are indicated by half arrows. (**B**) Quantitation of *SlHAM4* transcript in young leaves of *35S::SlHAM4* T_0 plants, normalized to *SlTIP41* as the reference gene. Error bars indicate ±SD over 3 technical replicates. (**C**) Pictures of mature tomato plants of M82 (wild type) and *35S::SlHAM4* genotypes grown under standard greenhouse conditions. Scale bars = 5 cm. (**D**) Schematic of the *SlHAM4* genomic

landscape including its regulatory sequences and neighbouring genes and the binary construct used for *slham4*$^{CR\Delta4}$ transformation. (**E**) Genotyping transgenic plants by PCR. Genomic DNA was isolated from mature leaves of transgenic plants and used for PCR using specific primers designed to specifically amplify the wild-type *SlHAM4* gene (top panels) and the corresponding transgenes (bottom panel; relative positions of used primers are indicated by half arrows in (**D**), respectively.

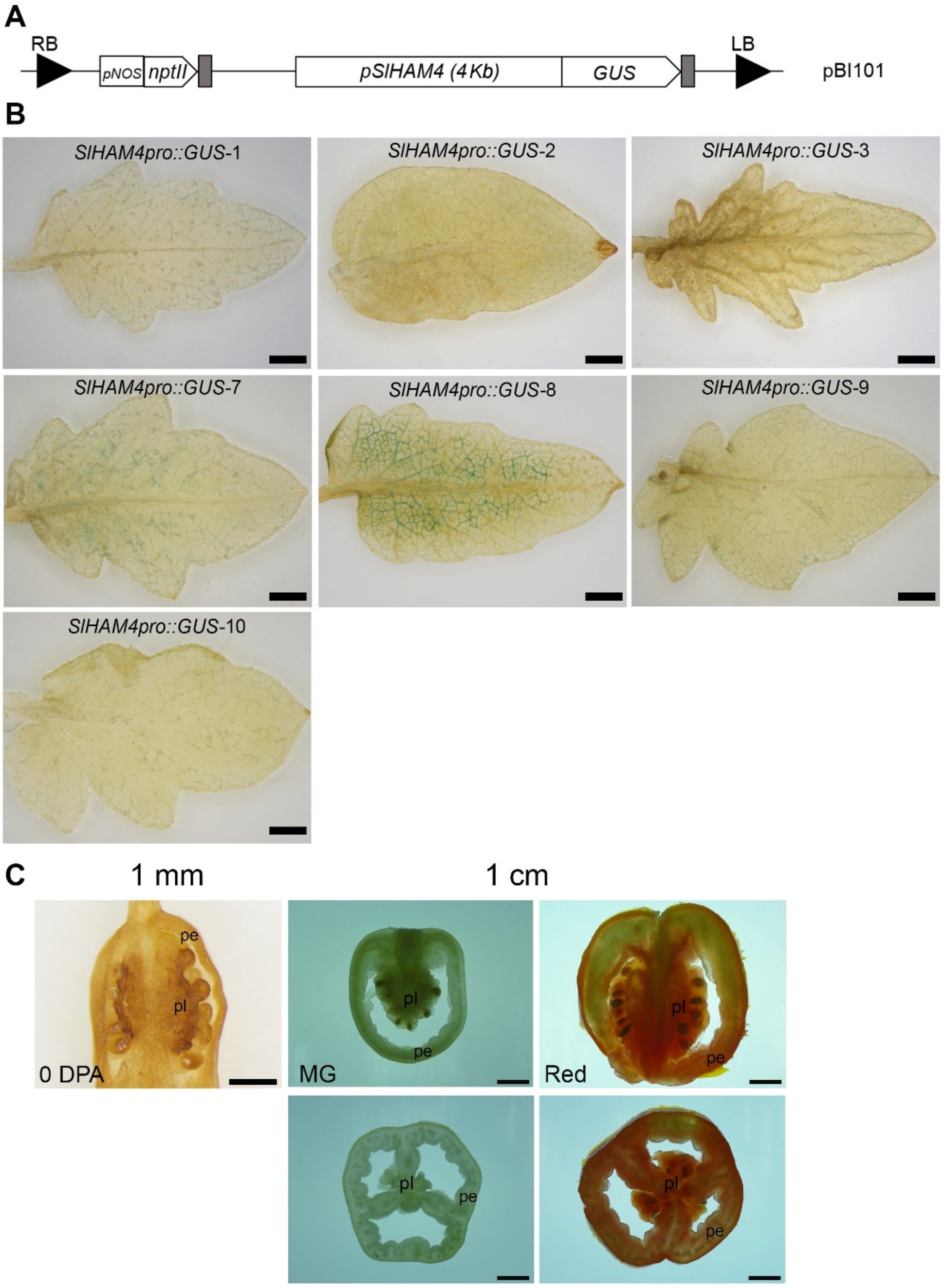

Figure S4. Analysis of T0 *SlHAM4* GUS reporter plants. (**A**) Schematic representations of the binary construct used for M82 transformation. (**B**) Representative pictures of GUS-stained leaflets of T0 transgenic *SlHAM4pro::GUS* plants. Scale bars = 1 mm. (**C**) Representative pictures of GUS-stained wild type M82 manual longitudinal (top panel) and cross (bottom panel) sections of anthesis ovary (0 DPA), mature green (MG) and red fruits. Scale bars = ovary (1 mm), Fruit (1 cm).

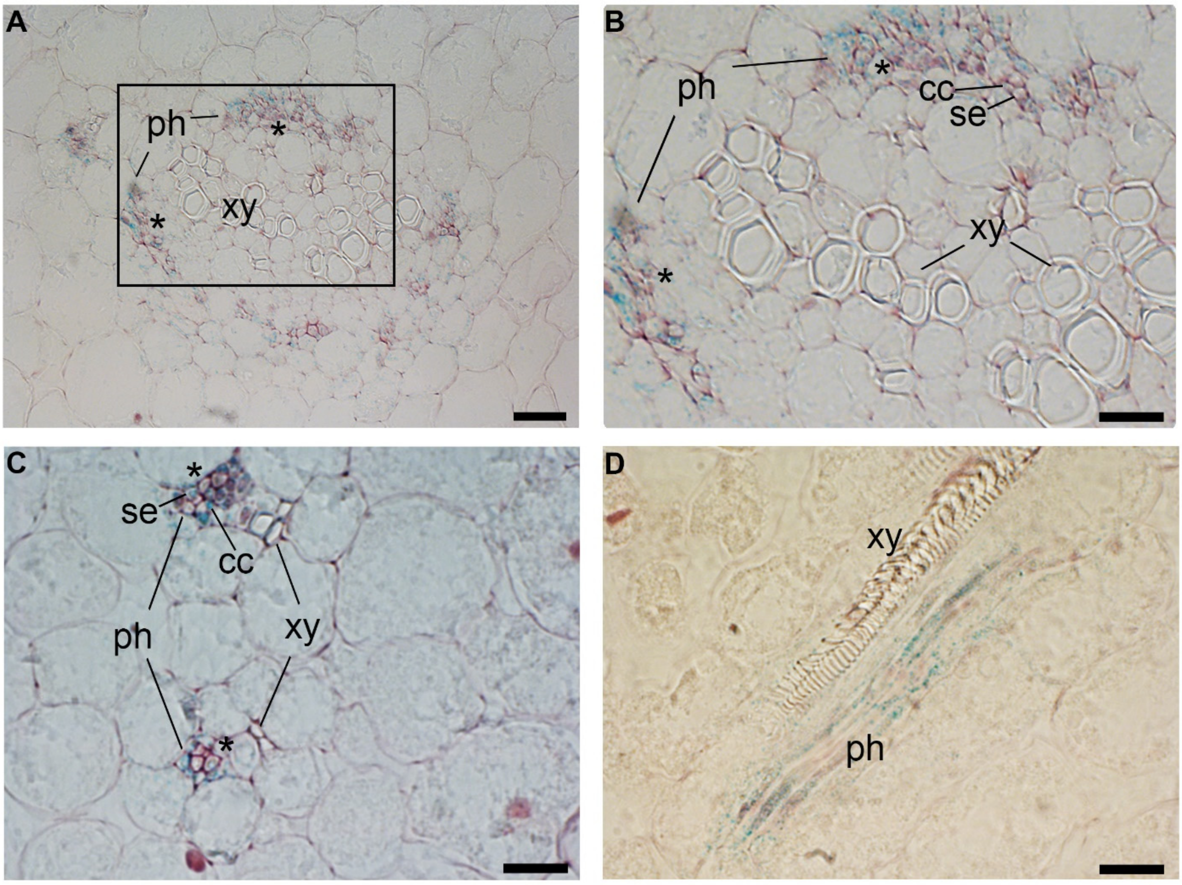

Figure S5. Histological analysis of the *SlHAM4pro::GUS-8* cotyledon vasculature region. (**A**) Cross-section of vasculature midvein. (**B**) Magnified view of the histological section outlined in **A** by a black box. Cross- (**C**) and longitudinal- (**D**) sections of minor veins. Asterisks mark GUS stained phloem bundle cells. Scale bars: 20 µm (**A**), 10 µm (**B** to **D**). se, sieve element cell; cc, companion cell; ph, phloem; xy, xylem.

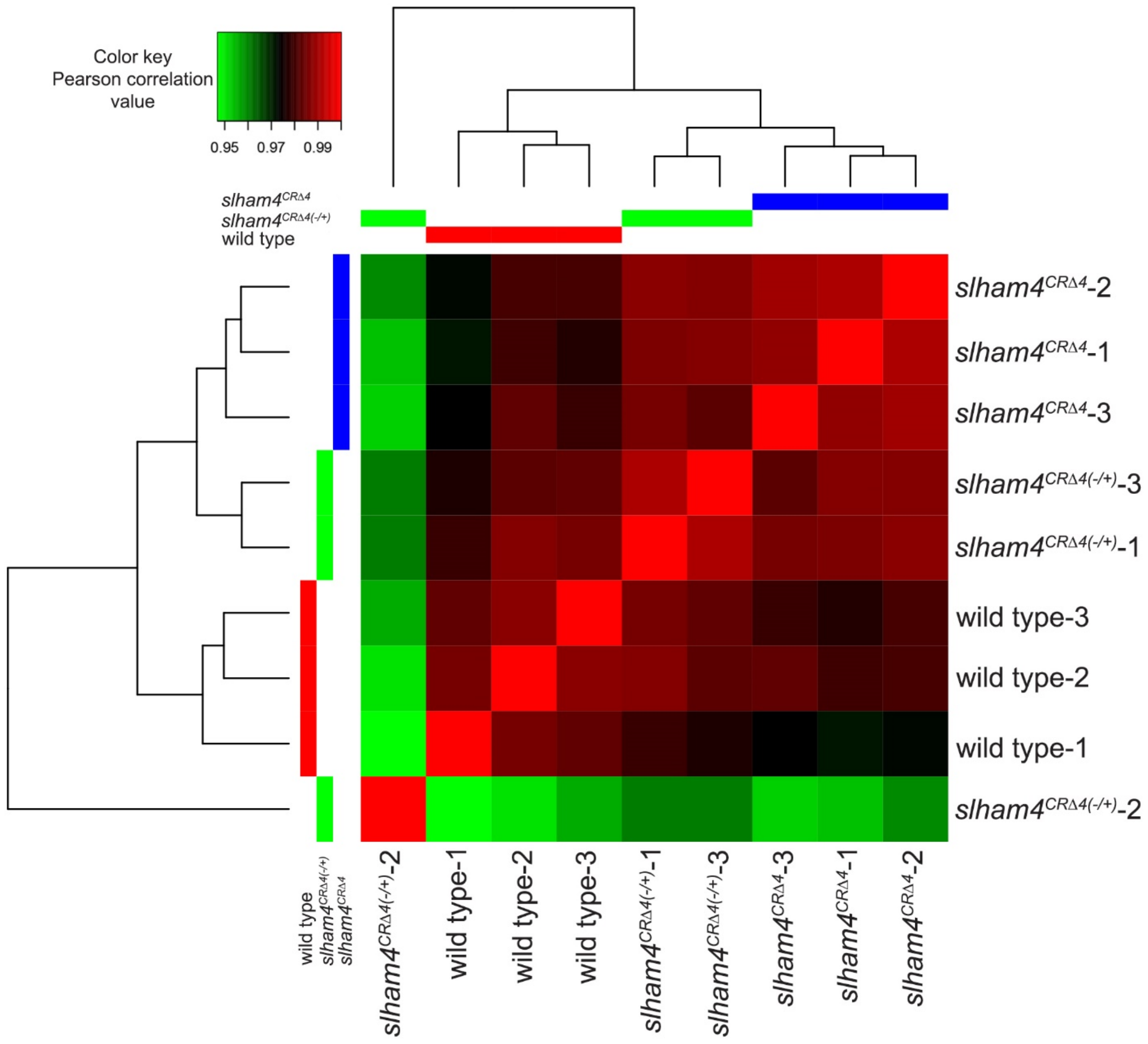

Figure S6. Heatmap of hierarchically clustered Pearson correlation matrix for gene expression in wild-type and *SlHAM4* mutant samples. Gene expression values are based on CPM (counts per million mapped reads) $\log_2$ transformed for each pair of samples. The three biological replicates of wild-type, *slham4*$^{CR\Delta4(-/+)}$ and *slham4*$^{CR\Delta4}$ are colored by red, green and blue, respectively. Note that *slham4*$^{CR\Delta4(-/+)}$-2 is separated from the other *slham4*$^{CR\Delta4(-/+)}$ biological replicates. The Heatmap and Pearson correlation were computed by R software.

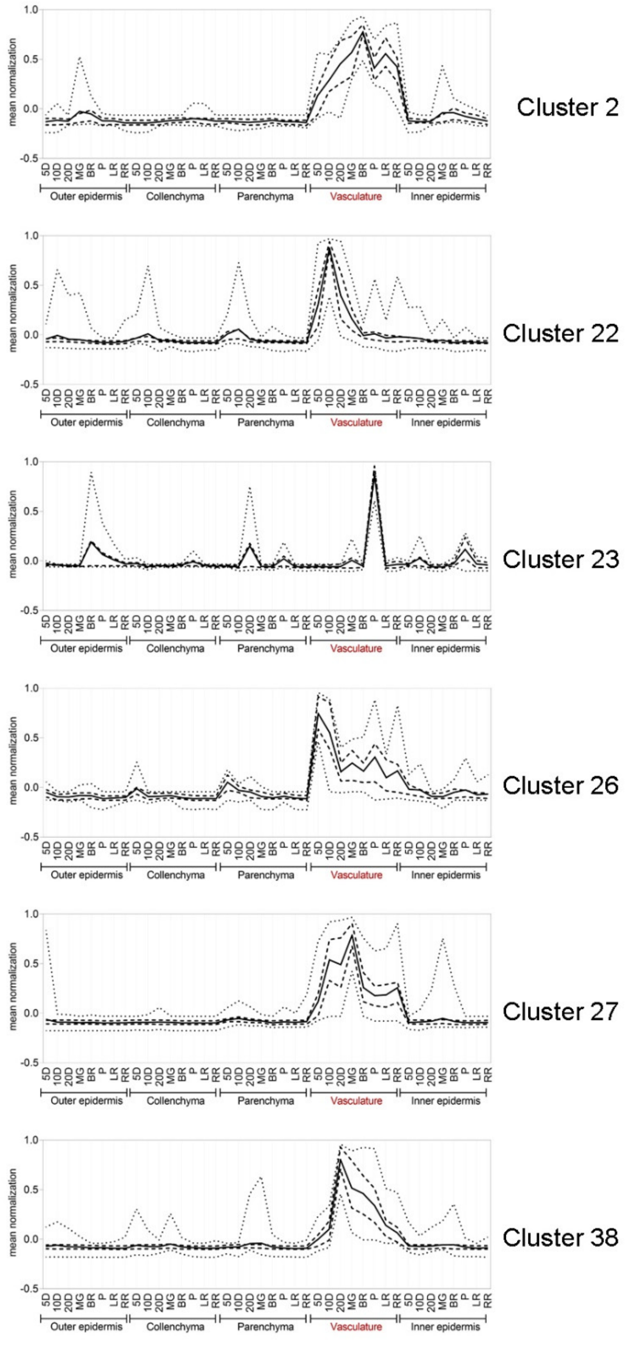

Figure S7. Expression profiles of indicated cluster cDEGs in fruit pericarp tissues based on TEA database data [3]. Cluster-wide average expression is plotted with solid lines, first and third quartiles by dashed lines, and maximum and minimum by dotted lines.

References

1 Zouine M *et al.* TomExpress, a unified tomato RNA-Seq platform for visualization of expression data, clustering and correlation networks. *Plant J* 2017; **92**: 727–735.

2 Waese J *et al.* ePlant: Visualizing and Exploring Multiple Levels of Data for Hypothesis Generation in Plant Biology. *The Plant Cell* 2017; **29**: 1806–1821.

3 Fernandez-Pozo N *et al.* The Tomato Expression Atlas. *Bioinformatics* 2017; **33**: 2397–2398.
